# Marine macroalgae as sources of protein and bioactive compounds in feed for monogastric animals

**DOI:** 10.1002/jsfa.9143

**Published:** 2018-06-30

**Authors:** Margareth Øverland, Liv T Mydland, Anders Skrede

**Affiliations:** ^1^ Department of Animal and Aquacultural Sciences Faculty of Biosciences, Norwegian University of Life Sciences Aas Norway

**Keywords:** marine macroalgae, nutritional value, bioactive components, feed, monogastric animals, biorefinery processing

## Abstract

Marine macroalgae are considered as promising sustainable alternatives to conventional terrestrial animal feed resources. The advantages include high growth rate, potential cultivation in saltwater, and no occupation of arable land. Macroalgae are broadly classified as brown (*Phaeophyta*), red (*Rhodophyta*) and green (*Chlorophyta*) algae, and are a diverse group of marine organisms. The nutritional value of macroalgae is highly variable. The protein and essential amino acid content can be low, especially in brown species, and indigestible polysaccharides adversely affect the energy value. Optimal use of macroalgae in feeds requires suitable processing, and biorefinery approaches may increase protein content and improve nutrient availability. Macroalgae are rich in unique bioactive components and there is a growing interest in the potentially beneficial health effects of compounds such as laminarin and fucoidan in different macroalgal and macroalgal products. This review summarizes current literature on different aspects of the use of macroalgae as sources of protein and health‐promoting bioactive compounds in feed for monogastric animal species. © 2018 The Authors. *Journal of The Science of Food and Agriculture* published by John Wiley & Sons Ltd on behalf of Society of Chemical Industry.

## INTRODUCTION

Food security is a great challenge, considering the rapidly growing global population and upwardly trending standards of living.^1^, ^2^ Increasing competition for land, water and energy, and fully exploited capture fisheries emphasize the urgent need for sustainable feed ingredients developed from under‐utilized renewable natural resources that do not compete with human food. The use of different marine macroalgae (seaweed) as a supplementary feed resource in animal production has a long history.^3^, ^4^, ^5^, ^6^ The advantages over terrestrial biomass include high growth rate, potential cultivation in saltwater, and no requirements for arable land or industrial fertilization.

Macroalgae contain varying levels of nutrients depending on species, season of harvest, geographic origin, and environmental conditions.^7^, ^8^, ^9^, ^10^, ^11^, ^12^ The protein and nutritionally essential amino acids content can be rather low and variable, especially in brown macroalgae, when considered against the amino acid requirement of most aquacultural and terrestrial animal species. The challenges of using macroalgae in animal feed include the high content of recalcitrant polysaccharide components such as alginates and carrageenans, which are not digested to any extent by monogastric animal species.^13^ This reduces the nutritionally available energy content of macroalgae and most algae‐derived products. In the early 2000s, the complex carbohydrates in macroalgae were recognized as having a prebiotic effect when used at low levels to supplement animal diets.^3^ Marine macroalgae are rich in bioactive compounds that can be converted to a variety of secondary metabolites with a broad spectrum of biological activities.^14^, ^15^, ^16^, ^17^ In the future, bioactive compounds with documented beneficial effects may facilitate increased commercial use of macroalgal products as feed ingredients.

Recent research findings have revived interest in marine macroalgal biomass as a potentially sustainable feedstock for production of feed ingredients for monogastric aquacultural and terrestrial livestock. This may uncover attractive possibilities for incorporating macroalgae indirectly into the human food chain. The objective of the present review is to provide an overview of the potential of marine macroalgae as a source of protein and bioactive compounds in feed for monogastric terrestrial and aquacultural animals, mainly emphasizing recently published data.

## CHEMICAL COMPOSITION OF MACROALGAE

Marine macroalgae are a diverse group of multicellular, plant‐like protists that can be classified into brown (*Phaeophyta*), green (*Chlorophyta*) and red (*Rhodophyta*) algae. The pigment responsible for the brown color of *Phaeophyta* is fucoxanthin, the red color of *Rhodophyta* comes from phycobilins, and several pigments (e.g., chlorophyll a and b, carotenes, and xanthophylls) are responsible for the green color of *Chlorophyta*.^18^ A brief summary is given in Table 1. The chemical composition of macroalgae varies considerably between species and with season of harvest, growth habitat, and environmental conditions. Even within a small geographic area, growth rate and chemical composition may vary depending on, e.g., harvest season,^19^ sunlight,^20^ salinity,^11^, ^21^ depth in the sea^22^ local water currents, or closeness to aquacultural plants.^23^ Reported ranges in proximate composition of brown, green, and red macroalgae are shown in Table 2.

**Table 1:** Brief summary of differences between groups of marine macroalgae^24^, ^25^, ^26^, ^27^, ^28^

	Brown macroalgae	Green macroalgae	Red macroalgae
Type of cell‐wall	Double	Single	Double
Type of chlorophyll	a, c	a, b	a
Main pigments	fucoxanthin, violaxanthin, β‐carotene	lutein, zeaxanthin violaxanthin neoxanthin, β‐carotene	lutein, zeaxanthin, phycobilliproteins, β‐carotene

**Table 2:** Ranges of proximate composition of marine macroalgae^a^

Chemical constituent	Brown macroalgae^b^	Green macroalgae^c^	Red macroalgae^d^
Water, g kg^−1^ of wet biomass	610–940	780–920	720–910
Crude protein^e^	24–168	32–352	64–376
Crude lipids	3–96	3–28	2–129
Polysaccharides	380–610	150–650	360–660
Ash	150–450	110–550	120–422

a Values are in g kg^−1^ of DM unless otherwise specified.

b Values are for typical brown macroalgal species: e.g., *Laminaria*, *Saccharina*, *Fucus*, *Ascophyllum*, *Alaria*, *Pelvetia* and *Undaria* spp. reported in the literature.^6^, ^10^, ^13^, ^21^, ^26^, ^28^, ^29^, ^30^, ^31^

c Values are for typical green macroalgal species: e.g., *Ulva*, *Cladophora*, and *Enteromorpha* spp. reported in the literature.^5^, ^6^, ^13^, ^28^, ^30^, ^31^

d Values are for typical red macroalgal species: e.g., *Palmaria*, *Chondrus*, *Porphyra*, *Vertebrata*, and *Gracilaria* spp. reported in the literature.^5^, ^6^, ^10^, ^13^, ^26^, ^28^, ^29^, ^30^, ^31^

e All values for CP have been recalculated using the recommended nitrogen‐to‐protein factor of five.^32^

### Protein and amino acids

Comparing the protein content of macroalgae reported in different studies can be difficult owing to methodological differences. Nitrogen is found in proteins, nucleic acids, and several other organic compounds such as chlorophyll. In addition, macroalgae contain significant amounts of inorganic non‐protein nitrogen (NPN; e.g., ammonia, nitrate, and nitrite). Spectroscopic methods are often used for protein determinations,^33^, ^34^ but many proteins from macroalgae can be difficult to extract and contain several colored substances that may influence the measurements. For these reasons, analysis of nitrogen and the use of a macroalgal‐specific nitrogen‐to‐protein conversion factor has been recommended^32^, ^35^ because the traditional nitrogen‐to‐protein factor of 6.25 used for most food and feed ingredients leads to an overestimation of the protein level. For nutritional purposes, however, amino acid analysis of the macroalgae should be performed. The protein content of brown macroalgae is generally low (usually below 150 g kg^−1^ of dry matter (DM)), whereas green macroalgae, and especially red macroalgae, have a higher protein content on a DM basis.^35^, ^36^ Some red macroalgae, such as *Porphyra* spp., have protein levels comparable to soybean meal, for example.^5^

**Figure 1:**
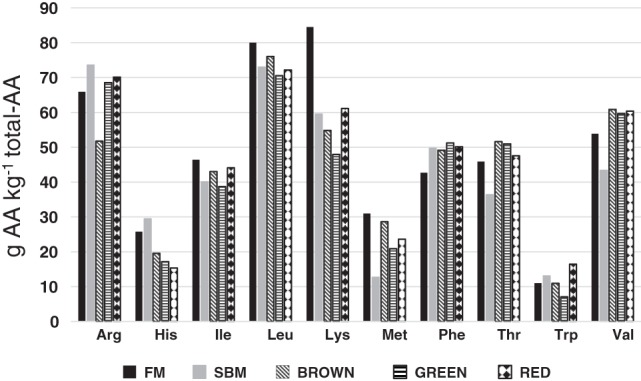
Typical essential amino acid (EAA) profiles of fishmeal (FM), soybean meal (SBM) and brown, green, and red marine macroalgae. Values are averages for the most common macroalgal species reported in the literature,^6^, ^10^, ^26^, ^29^, ^36^, ^37^, ^38^, ^39^, ^40^, ^41^ expressed as g AA kg^−1^ of total AA for each EAA.

Many species have an essential amino acid (EAA) to total AA (TAA) ratio of > 450 g EAA kg^−1^ of TAA.^4^, ^10^ A comparison of the average relative proportions of EAA in fishmeal, soybean meal, and brown, green, and red macroalgae is presented in Fig. 1. Compared with fishmeal, the lysine proportion is lower in macroalgae as a whole, but is usually higher in red than in brown and green species. Many macroalgal species are low in histidine, but the methionine content can be relatively high in many species. Macroalgae usually contain high levels of glutamic acid, which is present in both free and protein‐bound form,^10^ and contributes to the typical taste (umami) of macroalgae. Macroalgae also contain a number of bioactive amino acids and peptides (e.g., taurine, carnosine, and glutathione).^26^

### Polysaccharides

The large and morphologically diverse group of marine macroalgae contains many different complex carbohydrates and polysaccharides (Table 3). Brown macroalgae mainly contain alginates, sulphated fucoidans, and laminarin; green macroalgae contain xylans and sulphated galactans (ulvan); and red macroalgae contain agars, carrageenans, xylans, sulphated galactans, and porphyrans.^3^ The cell walls of marine macroalgae lack lignin, although ‘lignin‐like’ compounds and true lignin have been reported in some species.^42^, ^43^ In contrast to terrestrial plants, where lignin is important for rigidity, the cell walls of macroalgae are more flexible. The main structural components are alginate and fucoidan in brown, xylan and ulvan in green, and carrageenans in red macroalgae.^26^ The main storage components are laminarin in brown algae and floridean starch (amylopectin) in green and red species. Another main difference from cell walls in terrestrial plants is the presence of many uncommon polysaccharides that can be, e.g., sulphated, methylated, acetylated, or pyruvylated.^44^, ^45^, ^46^, ^47^ Compared with terrestrial plants, marine macroalgae have similar or higher levels of dietary fiber. They also contain other plant components like lignin. Since dietary fiber is not digested in the small intestine, it reaches the large intestine or colon where it can be partially or fully fermented.^26^, ^48^ The average total dietary fiber content can vary from 100 to 690 g kg^−1^ of DM. In red and green macroalgae, the soluble fiber fraction ranges from 520 to 560 g kg^−1^ of total fiber, but the soluble fiber content of brown algae is usually higher.^49^ Finally, macroalgae can also contain sugar alcohols such as mannitol. In fact, especially in some brown species, the mannitol content can be up to 25% of the dry weight.^26^

**Table 3:** Description and content range of carbohydrates in marine macroalgae^a^

Description/chemical constituent^b^	Brown macroalgae	Green macroalgae	Red macroalgae
Types of polysaccharide	alginate, laminarin, fucoidan (sulphated), cellulose, mannitol	ulvan (sulphated), mannan, galactans (sulphated), xylans, starch, cellulose, lignin	carrageenans (sulphated), agar (sulphated), glucans (floridean starch), cellulose, lignin, funoran
Types of monosaccharide	glucose, galactose, fucose, xylose, uronic acid, mannuronic acid, guluronic acid, glucuronic acid	glucose, mannose, rhamnose, xylose, uronic acid, glucuronic acid	glucose, galactose, agarose
Total fiber	170–690	290–670	100–590^c^
Soluble fiber	257–380	170–240	80–370^c^
Insoluble fiber	47–400	160–190	80–270
Specific polysaccharides			
Agar			210–420
Carrageenans			220–710
Alginate	140–400		
Alginic acid	170–330		
Fucoidan	20–200		
Laminarin	0–300		
Porphyran			480
Ulvan + xylan		400–550	
Floridean starch			250–420
Mannitol	20–250		
Lignin		30	

a Values are in g kg^−1^ of DM.

b Values are those reported for typical brown, green and red macroalgal species.^6^, ^13^, ^26^, ^28^, ^44^, ^47^, ^50^, ^51^, ^52^, ^53^, ^54^, ^55^, ^56^, ^57^

c Carrageenans are classified as soluble fibers; therefore, for some species with very high carrageenan levels, the fiber content can be higher than reported here. Soluble fiber analyses were not reported.^56^

### Lipids, phytochemicals and secondary metabolites

Marine macroalgal species have low lipid content (usually below 40 g kg^−1^ of DM), but the proportion of long‐chained polyunsaturated fatty acids (LC‐PUFA) is relatively high.^10^, ^30^, ^51^ As for other aquatic species, the content of PUFA is generally higher in those living in cold water,^58^ and will therefore be affected by environmental factors. The proportion of eicosapentaenoic acid (EPA; C20:5n3) can be well above 50% of total fatty acids,^59^ while the proportion of docosahexaenoic acid (DHA; C22:6n‐3) is lower and is only observed in some species.^13^, ^25^, ^30^, ^58^, ^60^ Lipid membranes also contain sterols; the main sterol in brown macroalgae is fucosterol (up to 97% of the total sterol content).^25^, ^26^

Macroalgae contain a wide range of organic compounds,^25^, ^26^, ^51^ that can be divided into polar phenols or phenol derivatives (e.g., phlorotannins and phloroglucinols) and non‐polar (unsaponifiable) compounds (e.g., sterols, tocopherols, triterpenes, and pigments). The phenols content of brown macroalgae is variable but can be considerable (< 10–140 g kg^−1^ of DM)^26^ compared with that of red and green macroalgae. The highest phlorotannin content is found in species within the genera *Ascophyllum*, *Fucus,* and *Sargassum*.^26^ Although many of these compounds exhibit a wide range of biological and pharmacological activities, they can also be considered anti‐nutritional factors. Other important metabolites in brown macroalgae include terpenes, bromophenols, and oxylipins.^25^, ^61^ In addition, macroalgae can also contain considerable amounts of tocopherols with strong antioxidative effects. According to Belghit *et al*.,^25^ brown macroalgae contain relatively large amounts of α‐, β‐, γ‐, and δ‐tocopherols, while red and green algae contain detectable levels of α‐tocopherol, with only traces of the other tocopherols.

### Minerals

Marine macroalgae are known for their high mineral content, and have traditionally been used as a mineral supplement for farm animals.^3^ The ash fraction can be as high as 550 g kg^−1^ of DM (Table 2), but for most species, the ash content is in the range of 200–350 g kg^−1^ of DM. Although macroalgae are rich in nutritionally important minerals such as iodine, potassium, calcium, magnesium, phosphorus, iron, and zinc, little is known about their bioavailability.^3^, ^6^, ^51^ Macroalgae can also accumulate large amounts of heavy metals, and the high levels of arsenic, lead, cadmium, and other heavy metals in some species can limit their use in animal feeds. However, the bioavailability of these metals is important in determining the toxicity risk,^31^, ^62^ and for many macroalgal species, the levels of available heavy metals are naturally below food and feed safety limits.^26^ A further important consideration is that low bioavailability of an undesirable component means high levels will be excreted in manure, which in turn will be applied to field crops. Also, the level of iodine in some macroalgal species, especially the brown species within *Laminaria* and *Saccharina* that can contain up to 12 000 mg kg^−1^DW,^26^ can limit their use in animal feed. Mineral content varies considerably between different species and phyla, and many other factors can have an influence, such as season and environmental conditions.^10^, ^26^, ^51^ Detailed information about the mineral composition of different macroalgae is beyond the scope of this review.

## PROCESSING FOR COMPOUND FEED APPLICATION

The harvested wet macroalgal biomass is bulky, watery, and heterogenous, and subject to rapid deterioration upon storage unless preserved by suitable methods. Processing for use in compound feed may be for preservation and homogenization, to retain or increase the concentration of essential nutrients and valuable bioactive compounds, to increase digestibility and functionality, or to remove potentially toxic substances. Large‐scale use of macroalgae as a feed resource requires a continuous supply of biomass. The growth and harvesting of macroalgae is usually seasonal; year‐round production processes therefore require preservation and long‐term storage. Except for the drying techniques, there is a paucity of research on efficient methods to preserve macroalgae.^63^ The early work of Black^64^ showed that lactic acid fermentation, commonly applied in grass conservation, is a promising preservation method for brown macroalgae. Adequate acidification of macroalgae by natural lactic acid fermentation is difficult because of the low content of rapidly fermentable carbohydrates, high buffering capacity, and low initial numbers of lactic acid bacteria.^63^ Previously, Uchida and Miyoshi^65^ reported that saccharification by cellulase and addition of a starter culture of lactic acid bacteria were beneficial when fermenting algae for food purposes. Initial pH reduction by acid addition may be beneficial by reducing unfavorable bacteria and stimulating lactic acid fermentation, as shown for ensiling pretreatment with hydrochloric acid.^66^ We are not aware of any published studies on the use of organic acids like formic acid and propionic acid, commonly used for grass preservation in bales or silos, in the preservation of macroalgae.

After drying and milling to a fine powder, macroalgae are traditionally used as seaweed meal in compound animal feed on a total biomass basis.^3^, ^67^ Oven drying by fossil energy is energy intensive and costly, and other technologies may be applied.^5^ Recent research has shown that the addition of dilute hydrochloric acid reduces the stickiness of the biomass, rendering it suitable for dewatering by screw‐pressing,^66^ and the pH reduction may facilitate efficient preservation. However, dewatering by screw‐pressing may result in losses of valuable water‐soluble components. The application of the entire biomass in a dry meal means that the nutritional value of the final product is greatly dependent on the macroalgal species, season, and other factors influencing chemical composition. In addition, the nutritional properties may depend on the drying methods employed, as was shown for certain brown species.^14^, ^68^

**Figure 2:**
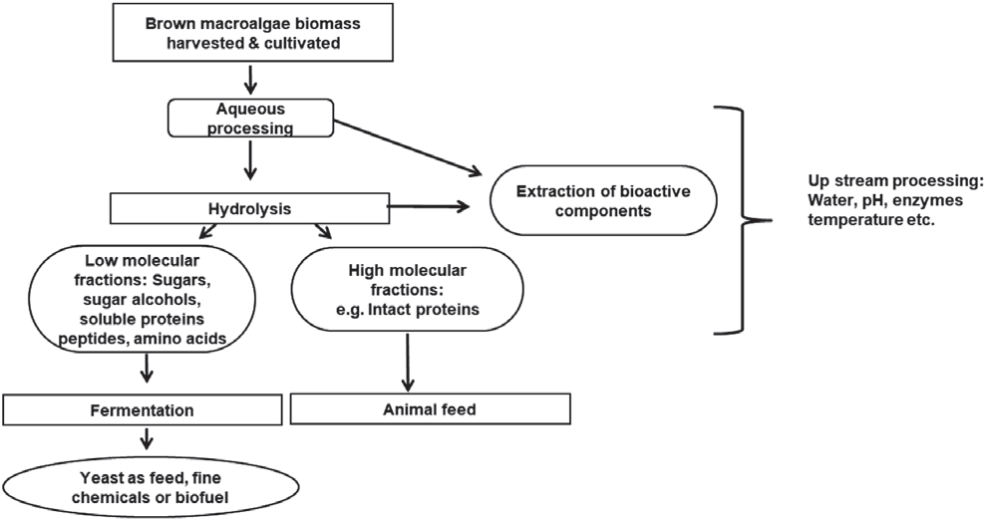
Conceptual flow chart of brown macroalgae processing (modified after Bikker *et al*.^41^) involving: (1) pre‐treatment of the biomass to remove salt and soluble components; (2) hydrolysis by acids or enzymes to convert macroalgal biomass to soluble and insoluble fractions; (3) fermentation of sugars, sugar alcohols, soluble protein and other nutrients to produce single‐cell proteins such as yeast; (4) extraction of bioactive compounds; and (5) direct extraction of proteins from the biomass.

Potential applications of macroalgal products in a cascading biorefinery model may be as protein sources with increased digestible amino acids and energy content, or as extracted bioactive compounds for high‐value applications as feed additives at low levels (Fig. 2). Protein concentration in macroalgal products can be increased by efficient extraction methods.^69^, ^70^, ^71^, ^72^ Possible methods include conventional as well as novel processing technologies such as enzyme‐assisted or microwave‐assisted extraction, pressurized liquid extraction, supercritical fluid extraction, and pulsed electric field.^5^, ^73^ The extraction of protein from macroalgae is made challenging by the complex polysaccharide cell wall and extracellular matrix, which is somewhat species dependent. Hydrolysis with a mixture of cellulase and xylanase increased the yield of protein extraction in *Palmaria palmata*.^72^ Other studies on protein extraction from *P. palmata*, using osmotic shock, high share force, and alkaline and polysaccharide treatments have shown increased protein recovery, but may be economically infeasible owing to the high enzyme:substrate ratio required.^74^ Since the polysaccharide composition varies among macroalgal species, the enzyme cocktail must be adapted for each algal species.

The digestibility of macroalgal proteins is inhibited owing to their entrapment in the cellular matrix,^13^ and may be improved by methods that liberate them by breaking down polysaccharides. Interactions with poorly accessible soluble polysaccharides such as xylan and carrageenan also reduce protein digestibility.^29^, ^75^ Bikker *et al*.^41^ showed that simulated *in vitro* ileal nitrogen digestibility was increased from 79.9% in intact *Ulva lactuca* to 84.7% in the extracted fraction, presumably through release of cell‐wall‐bound or encapsulated protein during pretreatment hydrolysis. Fermentation may also increase protein digestibility by degradation of insoluble fiber.^75^

Feed protein production from macroalgae may be increased by conversion of organic constituents like carbohydrates and non‐protein nitrogen into proteins by fermentation. Pretreatment by milling and enzymatic saccharification with cellulases, lamarinases, and alginate lyases efficiently releases fermentable sugars from brown macroalgae like *Laminaria digitata* and *Saccharina latissima*.^76^, ^77^, ^78^ This implies that macroalgal carbohydrates can be used as alternative carbon sources in fermentation processes to replace conventional carbohydrate sources like simple sugars. The chemical composition of the macroalgal biomass is complementary to lignocellulosic biomass,^79^ which may facilitate yeast production by cofermentation in a biorefinery approach. The macroalgae can supply essential nutrients like nitrogen and minerals that are lacking in lignocellulosic biomass. This may create possibilities for utilization of the indigestible polysaccharides as well as the nutritionally useless non‐protein nitrogen and mineral components in macroalgae. To our knowledge, there are no published scientific reports on yeast production by cofermenting macroalgae and lignocellulosic biomass.

Overall, efficient preservation, dewatering, and increased protein concentration may be necessary to support the inclusion of macroalgae as a sustainable protein source in compound animal feed. Downstream processing by biorefinery approaches has the potential to create feed for monogastric animals as a value‐added product from macroalgae. A number of protein extraction methods have been applied on a laboratory scale, but many studies lack detailed information on extraction procedures, and economic feasibility may be an obstacle. Large‐scale industrialization of production of nutritionally well‐defined animal feed products may require improved low‐cost separation technologies or fermentation procedures to convert sugars from complex macroalgal polysaccharides and non‐protein nitrogen into yeast protein.

## EFFECTS ON GROWTH PERFORMANCE, PROTEIN UTILIZATION AND CARCASS COMPOSITION

### Green macroalgae

Species of green algae sea lettuces (*Ulva* genus) have been the subject of several animal experiments. Intact biomass from *Ulva* spp. is relatively rich in protein and has potential as an alternative protein source in animal feed.^80^ In studies with broiler chickens, Abudabos *et al*.^81^ fed up to 30 g kg^−1^ of intact *U. lactuca* (sun‐dried for 3 days and then oven‐dried at 60 °C for 72 h to 231 g kg^−1^ of crude protein (CP) and 893 g kg^−1^ of DM) with no significant differences in growth rate, feed intake or feed conversion ratio. However, they observed positive effects on dressing yield and percentage of breast meat, and a reduced level of abdominal fat. The latter effects were potentially related to the slightly higher levels of protein and methionine in the diet containing the highest level of *U. lactuca*. Dry powder of the green macroalga *Ulva* (*Enteromorpha*) *prolifera* increased feed intake and daily weight gain, and improved feed conversion ratio when fed at levels up to 40 g kg^−1^ in diets for broiler chickens.^82^ The researchers reported that adding *U. prolifera* powder to the diets decreased abdominal and subcutaneous fat, improved breast meat quality, and increased amylase activity in the duodenal contents of the chickens. Studies by Ventura *et al*.^83^ showed that oven‐dried *Ulva rigida* containing 206 g kg^−1^ of CP on a DM basis reduced the metabolizable energy content of diets and had negative effects on growth performance when fed to chickens from 10 to 20 days of age. The negative effects were attributed to the presence of indigestible polysaccharides, suggesting that enzyme addition might have improved the results. In contrast, up to 100 g kg^−1^ of sun‐dried and ground *U. rigida* containing 295 g kg^−1^ of CP in DM had no negative effects on growth performance, protein digestibility and retention, and whole‐body composition when substituted for dietary fish protein hydrolysate in feed for European sea bass (*Dicentrarchus labrax*) juveniles.^84^ Marinho *et al*.^85^ studied dietary substitution of LT fishmeal in pelleted diets for Nile tilapia juveniles with 100, 150, and 200 g kg^−1^ of oven‐dried and milled integrated multi‐tropic aquaculture‐cultivated *Ulva* spp. (50:50% mixture of *U. rigida* and *U. lactuca*) containing 291 g kg^−1^ of CP in DM. They concluded that up to 100 g kg^−1^ of *Ulva* spp. meal could be fed without compromising growth performance, protein utilization, or protein retention, although dietary CP decreased and ash increased with dietary inclusion of *Ulva* spp. However, this level of *Ulva* spp. meal significantly increased FCR and reduced body protein content. Increasing the substitution to 150 or 200 g kg^−1^ of *Ulva* spp. meal resulted in further increases in FCR and substantially reduced final body weight and specific growth rate. Wassef *et al*.^86^ fed gilthead seabream (*Sparus aurata* L.) pelleted isonitrogenous diets containing 50, 100, or 150 g kg^−1^ of *U. lactuca* meal dried at 60 °C and containing 174 g kg^−1^ of CP in the DM. The results showed that the best growth performance, feed conversion ratio, protein efficiency ratio, and survival were obtained by feeding *U. lactuca* at 50 g kg^−1^. However, all diets containing *U. lactuca* appeared to stimulate feed intake and tended to give higher weight gain and specific growth rate than the fishmeal‐based control diet. Feeding of a moist‐type diet containing 5 g kg^−1^ of air‐dried and pulverized *Ulva pertusa* (corresponding to about 12% of DM) to fingerling red sea bream (*Pagrus major*) increased body weight gain, feed efficiency, and muscle protein deposition.^87^ The most pronounced positive effects in the latter study, however, were obtained for *Porphyra yezoensis* and *Ascophyllum nodosum* meals. Overall, the results show the suitability of algal meals in this type of diet for red sea bream.

The knowledge provided by studies on green macroalgal meals as feed ingredients shows their potential as substitutes for conventional feed protein sources, but the responses in growth performance have been variable. This is not surprising considering the great variation in nutritional content of the different products owing to factors such as macroalgal species, season of harvest, geographical origin, processing, and experimental design.

### Red macroalgae

The red macroalgae show a high level of biodiversity, but few of the described species have been studied as ingredients of diets for monogastric animals. Several red macroalgae are rich in protein and may be used in intact dried form as protein sources in formulated animal feed, although protein digestibility for the intact algae may be low.^88^, ^89^ Wan *et al*.^90^ fed Atlantic salmon (*Salmo salar*) diets formulated with 50, 100, and 150 g kg^−1^ of dried and milled *P. palmata* containing 220 g kg^−1^ of DM, basically replacing fishmeal and cornstarch in isonitrogenous, isolipidic, and isoenergetic (gross energy) diets. The results showed no difference in growth rate or feed conversion ratio across algal and control diets. It was concluded that *P. palmata* can be a suitable component in feed for Atlantic salmon. Studies of the effects on the quality of fresh and cooked fish fillets when *P. palmata* was included in diets for Atlantic salmon showed the yellow/orange color was enhanced through deposition of algal pigments, and dietary inclusion of 50 g kg^−1^ of *P. palmata* may improve overall acceptability without negatively impacting texture, odor, or oxidation flavor.^91^

Red macroalgae of the genus *Porphyra*, e.g. *P. purpurea*, *P. yezoensis*, and *P. dioca*, have been studied as protein sources in diets for different fish species. Davies *et al*.^92^ included *P. purpurea* meal containing 250 g kg^−1^ of CP at 165 and 330 g kg^−1^ in isonitrogenous and isoenergetic diets for the omnivorous thick‐lipped grey mullet (*Chelon labrosus*), replacing fishmeal. The results showed that body weight gain, specific growth rate, feed efficiency, and protein efficiency ratio as well as net protein utilization decreased with increasing *P. purpurea* inclusion levels. However, carcass analyses revealed that final protein, lipid, and ash contents in fish fed algae were not significantly different from the control fish fed a diet without algae.

In studies with the Atlantic cod (*Gadus morhua*), Walker *et al*.^93^ fed isonitrogenous and isoenergetic diets containing 55 and 110 g kg^−1^ of *Porphyra* spp. (> 90% *P. umbilicalis*) meal containing 321 g kg^−1^ of protein, in principle replacing fishmeal. There were no significant differences among treatments in growth performance, and the authors concluded that *Porphyra* spp. provide a suitable fishmeal replacement in diets for juvenile Atlantic cod. However, the interpretation of the results may be questioned because dietary *Porphyra* spp. inclusion was combined with increased levels of blood meal and reduced protein from corn and wheat gluten.^93^ In studies with rainbow trout where dried and milled *P. dioca* was fed at 50, 100, and 150 g kg^−1^, replacing fishmeal and wheat starch in isonitrogenous and isolipidic diets, dietary inclusion of the algae had no significant effect on growth performance indicators such as weight gain, specific growth rate, feed conversion ratio, and protein efficiency ratio.^94^ Carcass analyses revealed only minor differences between treatments, but the flesh pigmentation of the rainbow trout turned from pinkish‐white in the control fish to pinkish‐orange to dark orange in the fish fed 150 g kg^−1^ of *Porphyra* spp.^94^ This indicates that natural pigments from *Porphyra* spp. may enhance its potential for inclusion in feed for salmonids by reducing the need for artificial colorants. Studies with another red alga, *Gracilaria vermiculophylla*, in diets for rainbow trout showed that inclusion of 100 g kg^−1^ of *Gracilaria* meal increased skin carotenoid content but resulted in reduced growth performance and protein efficiency ratio.^95^ However, 50 g kg^−1^ of *G. vermiculophylla* could be fed without compromising growth performance and nutrient utilization. Other species of the genus *Gracilaria*, e.g. *Gracilaria bursa‐pastoris* and *Gracilaria cornea*, have been studied as ingredients in pelleted diets for European sea bass juveniles,^84^ replacing high‐quality fishmeal. The protein contents of the sun‐dried *G. bursa‐pastoris* and *G. cornea* were 302 and 110 g kg^−1^, respectively, and in particular, the inclusion of *G. cornea* reduced dietary protein and increased ash content. There were no adverse effects on growth performance at dietary inclusion levels of 100 g kg^−1^ for *G. bursa‐pastoris* and 50 g kg^−1^ for *G. cornea*, while 100 g kg^−1^ of *G. cornea* reduced growth rate and feed efficiency. Sotoudeh and Mardani^96^ fed a *Gracilaria pygmaea* meal to rainbow trout fry and reported improved growth performance with the inclusion of 60 g kg^−1^ of *G. pygmaea*, but reduced final body weight, specific growth rate, and protein efficiency ratio when the inclusion level was increased to 120 g kg^−1^. Studies with extruded isoenergetic, isonitrogenous, and isolipidic diets for the European sea bass showed that 75 g kg^−1^ of *Gracilaria* spp. or a mixture of 25 g kg^−1^ of *Gracilaria* spp., 25 g kg^−1^ of *Ulva* spp., and 25 g kg^−1^ of *Fucus* spp. had no negative effects on growth parameters.^97^

In studies with red sea bream, Mustafa *et al*.^87^ observed that dietary inclusion of 50 g kg^−1^ of *P. yezoensis* meal improved body weight gain, feed efficiency and muscle protein deposition. Similarly, Stadtlander *et al*.^98^ showed that inclusion of 150 g kg^−1^ of *P. yezoensis* Ueda meal in isonitrogenous and isoenergetic diets for intensively fed Nile tilapia, replacing fishmeal, improved growth rate, feed efficiency, and protein efficiency ratio, whereas there were no differences between fish fed the control and the 300 g kg^−1^ *P. yezoensis* diets. The authors indicate that the reasons for the growth‐promoting effect of the 150 g kg^−1^ *P. yezoensis* diet were unknown, and could not be explained by a superior amino acid profile.

Overall, the studies show the great potential of several red macroalgae as feed ingredients for fish, but few studies have been carried out with pigs and poultry. Many red algae are commonly used as components of human food, and high cost is a main reason for the paucity of research on their use as a protein source in diets for terrestrial farm animals. A general beneficial effect of low‐level supplementation in fish diets may indicate a positive effect of unidentified bioactive compounds. Conversely, a relatively low nutritional value might explain their deleterious effect on overall growth performance at high inclusion levels in some experiments.

### Brown macroalgae

The brown macroalgae are characterized by their large size and high productivity, and they are easily accessible in many locations, but the chemical composition of the whole biomass is not suitable for high inclusion rates in animal diets. The low levels of protein and metabolizable energy, and the high mineral content of intact brown seaweeds like *Laminaria* spp. and *A. nodosum*, prohibit their use as replacements for major protein sources such as fishmeal and soybean meal in formulated feed for monogastric animals. Jones *et al*.^99^ recorded weight loss in pigs fed 100 g kg^−1^ of a meal produced from *A. nodosum*, an alga that characteristically contains less than 100 g kg^−1^ of protein in the DM. Similarly, Whittemore and Percival^100^ concluded that the residue from *A. nodosum* after extraction of alginate was poorly digested and unsuitable as a protein and energy source for pigs. There are few published reports on the inclusion of brown macroalgae in formulated compound feed for fish. Costa *et al*.^101^ showed no effect of increasing levels of *A. nodosum* meal up to 20 mg kg^−1^ on body weight, but feed conversion ratio and carcass yield were improved.

Feeding of extracts from brown seaweed (*L. digitata*) containing laminarin and fucoidan may improve the quality and shelf life of pork, and reduce lipid oxidation in muscle tissue.^102^ This finding shows the potential for incorporation of macroalgal‐derived antioxidant components into human food through the animal diet, and suggests novel, well‐defined functional compounds are likely to be discovered in macroalgae. Instead of using intact brown macroalgae as lower‐value feed commodities, a preferable application may be as a higher‐value source of bioactive substances used at low levels to potentially improve growth performance and health, as discussed in the following section.

## HEALTH EFFECTS IN ANIMALS

Brown, red and green marine macroalgae are rich sources of structurally diverse bioactive components with valuable pharmaceutical and biomedical potentials^5^, ^14^, ^26^, ^103^ that could be exploited as functional health‐promoting ingredients in animal feeds. The bioactive components found in marine macroalgae depend on the species, but also on environmental factors such as geographical location, season, and harvest time.

Research has shown the effects of dietary supplementation with macroalgae or macroalgal extracts on the immune status and intestinal health of several monogastric farm animal species including pigs,^104^, ^105^, ^106^, ^107^, ^108^, ^109^, ^110^, ^111^, ^112^, ^113^ broiler chicken,^3^, ^114^, ^115^ and fish.^90^, ^97^, ^116^ Because of their health‐ and growth‐promoting effects, it has been suggested that bioactive components from macroalgae such as *Laminaria*‐derived laminarin and fucoidan can serve as alternatives to in‐feed antibiotics^105^, ^111^, ^117^ or as environmentally friendly alternatives to therapeutic dosages of zinc oxide in pig diets.^111^ Positive effects on animal health have been documented when feeding extracts from algal species, especially *Laminaria*, while few studies have evaluated the health effects of adding intact macroalgae to diets for monogastric animals. Dietary supplementation with *Laminaria* spp. or extracts containing laminarin and fucoidan to weanling pigs improved intestinal health,^105^, ^106^, ^109^, ^110^, ^111^, ^112^, ^118^ alleviated common problems occurring post‐weaning,^119^ and reduced post‐weaning diarrhea.^105^

Macroalgal extracts may enhance growth performance and gut health in part by altering gut architecture and thereby increasing the digestibility and absorption of nutrients, and by altering gut microbiota and/or modulating immune function and thus strengthening the gut barrier function.^105^, ^118^, ^120^ For instance, an increase in beneficial bacteria such as *Lactobacillus* spp. and * Bifidobacterium* in the gastro‐intestinal tract, and a decrease in the numbers of potentially pathogenic bacteria such as *Enterobacteria* have been reported when adding laminarin or fucoidan to piglet diets.^105^, ^106^, ^109^, ^111^, ^112^, ^117^, ^118^, ^120^ Also, increased villus height and villus height‐to‐crypt depth ratio in the small intestine, increased production of volatile fatty acids, and reduced pH in the hind gut have been reported^108^, ^110^, ^117^ Different modes of action of these components on gut health have been reported, however, which might be due to differences in the biochemical structures of the two compounds. Feeding piglets diets containing laminarin but not fucoidan increased expression of the nutrient transporters GLUT1, GLUT2, and SGLT1, which may partly explain the increase in nutrient digestibility and improved performance as these are responsible for transporting glucose from the lumen to the enterocytes and the bloodstream.^112^, ^120^ Also, the smaller molecular size of laminarin allows it to have a direct effect on gut mucosa or gut‐associated lymphoid tissue, which can strengthen the gut barrier function and enhance the immune function of the gut. For instance, dietary laminarin has been shown to increase the expression of genes involved in mucin production during weaning, such as *MUC2* in the colon, thus stimulating colonic mucosa.^121^ Laminarin is also taken up by the epithelial cells and Peyer's patches, and is presented to underlying dendritic cells to influence cytokine production and thereby improve gut health through an immunomodulatory effect. Supplementing diets with laminarin downregulated the expression of a panel of inflammatory cytokines in the colon and liver.^110^, ^120^ Downregulation of pro‐inflammatory cytokines may improve growth performance by providing more nutrients for growth by partitioning nutrients away from stimulating immune responses.^110^ In contrast, the larger molecular weight of fucoidan means it serves mainly as a source of rapidly fermentable carbohydrates that escape hydrolysis in the small intestine, and also exerts a prebiotic effect in the hind gut. As discussed by Reilly *et al*.,^118^ fucoidans are powerful antimicrobial agents: they inhibit the attachment of certain bacterial species in the gut and prevent the binding of *Enterococci* and *Streptococci* spp. to the extracellular matrix protein of the animal cells. Also, fucoidan has the ability to agglutinate certain bacterial species, inhibiting their attachment to epithelial cells and preventing them from colonizing the mucosal surface. These findings have been contradicted in other reports, however. For instance, McDonnell *et al*.^105^ reported that feeding a combination of laminarin and fucoidan reduced post‐weaning diarrhea, laminarin alone reduced fecal *Escherichia coli* counts and improved growth performance, and feeding fucoidan alone had no effect on gut health or growth performance. While feeding laminarin alone led to a reduction in *Enterobacterium* spp., when given with fucoidan, the level of *Enterobacterium* spp. in the proximal and distal colon increased.^117^ Also, supplementing the diet with either purified laminarin or fucoidan alone modified intestinal morphology and selected intestinal microbiota, although these effects were not observed when laminarin and fucoidan were offered in combination.^109^, ^110^ The results suggest that both laminarin and fucoidan have a positive effect on gut health, but laminarin has the added benefit of increasing the expression of *MUC* genes and nutrient transporter genes, and reducing proinflammatory cytokine gene expression. Taken together, these results suggest that inclusion of laminarin is more beneficial than fucoidan or the combination of the two supplements in diets for weaned pigs. However, the contradictory results when feeding laminarin and fucoidan warrant further study to better understand their mode of action on gut health.

An alternative approach to supplementing macroalgal extract to weaning pigs is through maternal supplementation. Recent studies have shown positive effects of supplementing sow diets with laminarin and fucoidan derived from *Laminaria* spp. during gestation and lactation on the gastrointestinal health and growth performance of weaned piglets.^107^, ^108^, ^122^ Dietary supplementation with a combination of laminarin and fucoidan to pregnant sows increased the IgG concentration in the colostrum and subsequently the serum concentration of IgG in suckling piglets. Lower fecal enterobacterial counts in sows at parturition and decreased *E. coli* counts in the suckling pigs were also reported, and the piglets had increased villus height and villus height‐to‐crypt ratio in the jejunum and ileum, and higher growth rate. The mechanism for the reduction in *E. coli* counts in suckling piglets and the immunomodulatory effect of maternal macroalgal extract supplementation could be mediated by mammary uptake of low‐molecular weight laminarin and its introduction to the suckling piglets' gastro‐intestinal tracts, but this was not measured. In addition, upregulation of tumor necrosis factor (TNF) and trefoil factor (TFF) mRNA expression in the ileum and colon suggests that macroalgal extract supplementation enhances the immune status of newly weaned piglets.^108^ The improvement in performance and health could also be partially explained by the reduction in expression of the pro‐inflammatory IL‐1α mRNA in the ileum, indicating that the macroalgal extract modulated pro‐inflammatory cytokines and immunity mediation and subsequent nutrient partitioning for normal growth and feed efficiency.^108^ Also, maternal laminarin supplementation following an *S. typhimurium* challenge resulted in improved growth rate, feed efficiency, and fecal scores, and increased production of VFA in the colon, while expression of IL‐22, a protein involved with maintenance of the mucosal barrier and tissue generation, was reduced.^113^

Extracts from *A. nodosum* have been extensively studied and have shown beneficial health effects in diets for pigs,^3^ but the effects depended on the levels used. Turner *et al*.^123^ reported that supplementing piglet diets with *A. nodosum* extract (0, 5, 10, and 20 g kg^−1^) improved growth performance, but had no beneficial effect on immune responses in the presence or absence of an *S. typhimurium* challenge. Supplementing growing/finishing pig diets with 3, 6, or 9 g kg ^−1^ of *A. nodosum* extract reduced coliform counts in the gastro‐intestinal tract and thereby improved gut health, but daily weight‐gain decreased linearly, possibly due to the presence of inhibitors such as phenolic compounds and alginate in the algal extract.^104^ However, when supplementing weanling diets with intact dried *A. nodosum* (2.5, 5, or 10 g kg^−1^)*,* no effect on gut bacterial population, morphology, plasma oxidative status, or growth performance was reported.^124^ In contrast, Dierick *et al*.^125^ reported a reduction in *E. coli* load in the stomach and small intestine, and a beneficial shift in the microbial population in the small intestine when supplementing piglet diets with 10 and 20 g kg^−1^ of intact *A. nodosum*.

Supplementing diets with macroalgal extracts has also been shown to improve the gastrointestinal health of broiler chickens. Sweeney *et al*.^115^ reported that supplementing diets for broiler chickens with purified laminarin or a mixture of laminarin and fucoidan extracted from *L. digitata* improved feed intake, the small intestinal architecture, and growth rate in the post‐hatch period, and upregulated the expression of key genes involved with immune responses. The improvement in growth performance could be attributed to increased palatability of the diet and/or increased nutrient digestion and absorption owing to the increased absorptive surface in the intestine. However, feeding a combination of laminarin and fucoidan had adverse effects on the birds' feed conversion ratio, suggesting that laminarin rather than a combination of laminarin and fucoidan has the potential to improve growth performance of broiler chickens post‐hatching. The authors suggested this could be because the laminarin and fucoidan mixture was less pure and might contain other compounds such as alginate and mannitol that are potential growth inhibitors. Extracts from *A. nodosum* have also been shown to affect the gut health when fed to broiler chickens. Evans and Critchley^3^ reported that feeding Tasco, an *A. nodosum* extract, to chickens resulted in a significant prebiotic effect. Supplementing broiler diets with 0.5 and 1.0 g kg^−1^ of *A. nodosum* extract exerted a positive effect on gut integrity and decreased the bacterial load in the cecum of 10‐day‐old chickens colonized with *C. jejuni,* but reduced growth performance.^114^ The research suggests that the extract from *A. nodosum* or *L. digitata* can improve growth performance by stimulating increased feed intake, increasing the uptake of nutrients from the lumen, and by stimulating the immune function and promoting a healthy gut microbiota. However, feeding a combination of laminarin and fucoidan appears to adversely affect growth performance, while feeding a purified laminarin alone appeared to be most efficient in improving growth performance and health, as also reported for pigs.

Macroalgae or extracts have received increasing attention as safe alternatives to prophylactic and therapeutic agents in diets for farmed fish to prevent economic losses related to infectious diseases. All three groups of macroalgae, red, green, and brown, have been shown to exhibit antimicrobial properties^116^ and inhibitory effects against fish pathogens^126^ *in vitro*. Limited information exists on the effect of dietary macroalgal supplementation on the health of farmed fish *in vivo*, however, although there appears to be increasing interest in the use of macroalgae as a bioactive component in functional feeds for fish. Health‐promoting effects include improved immunological responses, such as effects on lysozyme activity and increased complement pathway activity, increased antioxidant activity, and improved stress responses. Peixoto *et al*.,^97^ for instance, reported that supplementing diets for European sea bass with 75 g kg^−1^ of the red alga, *Gracilaria,* or a mixture of 75 g kg^−1^ of *Gracilaria* spp., brown *Fucus* spp., and green *Ulva* spp., may alter the metabolic rate, modulate the innate immune response, and cause antioxidant responses without compromising growth performance. The *Gracilaria* diet also resulted in increased glutathione S‐transferase, an enzyme responsible for removing reactive oxygen species (ROS), suggesting that macroalgal supplementation may protect fish from ROS. The immunostimulatory properties of macroalgae may depend on the inclusion rate: Peixoto *et al*.^97^ reported a decrease in the hemolytic capacity of the alternative pathway complement system with the inclusion of 75 g kg^−1^ of *Gracilaria* or 75 g kg^−1^ of a macroalgal mixture, while Araujo *et al*.^95^ reported an increase in the plasma alternative complement when supplementing diets for rainbow trout with 50 g kg^−1^ of *G. vermiculophylla*, whereas a decrease in the immune response occurred at a higher inclusion level of 100 g kg^−1^. In Nile tilapia, inclusion of 100 g kg^−1^ of meal from *U. rigida* and *U. lactuca* increased the alternative complement, while inclusion at 50 g kg^−1^ had no effect.^127^ In the same species, inclusion of 50 g kg^−1^ of *U. lactuca* and *Pterocladia capillacea* improved growth performance and nutrient retention as well as the stress response and survival rate after air exposure.^128^ In grouper (*Epinephelus coioides*), feeding diets containing 5 g kg^−1^ and 10 g kg^−1^ of laminarin improved growth rate and feed conversion ratio.^129^ In Atlantic salmon, diets containing 50 and 150 g kg^−1^ of *P. palmata* decreased serum activity of alanine transaminase, a biological indicator of liver health status, while there was no effect at 100 g kg^−1^.^90^ The research suggests that several macroalgal species, especially when used at low levels, provide health benefits when fed to fish and therefore have potential as ingredients of functional fish feed.

In general, these studies suggest that several macroalgal species and their extracts have beneficial health effects and potential as sources of bioactive compounds in feed for monogastric aquacultural and terrestrial livestock. However, reports of the effects on gut health of intact macroalgae and macroalgal extracts are inconsistent. This could be due to inhibitors in the intact macroalgae or the extracts, differences in bioactivity of compounds like laminarin or fucoidan from different macroalgal species, or differences in experimental design. In future work, attention needs to be paid to developing standard methods for extraction, isolation, and characterization of bioactive components in macroalgae as well as standardized methods to evaluate the impact of these on animal health in *in vivo* experiments.

## CONCLUSION AND FUTURE PERSPECTIVES

Macroalgae and macroalgal products are receiving increasing global attention as potentially sustainable ingredients in feed for monogastric aquacultural and terrestrial livestock. Macroalgae can be used as sources of protein and bioactive compounds in formulated feeds, and thereby be indirectly included in the human food chain. Earlier studies of macroalgae as whole biomass products in diets for monogastric animals produced inconsistent results. This may be partly due to variable and often inadequately defined macroalgal products, differences in basal diet composition, dietary inclusion levels, and ingredient replacement strategy, as well as differences in experimental protocols (e.g., conditions and response parameters). The levels of protein and potentially limiting essential amino acids in macroalgae vary greatly, and protein digestibility may be affected by species‐specific polysaccharides and phenolic compounds. It is therefore not feasible to generalize about the usefulness of whole macroalgae as a protein source, but many species have too little digestible protein to be attractive as alternative protein sources in animal feed. Studies have shown that protein concentration in macroalgal preparations can be increased by suitable extraction methods. There are, however, limited published data on the effects of processing on nutritional value and growth performance in different animal species. Future research efforts should be directed towards cost‐effective processing methods to increase the levels of biologically available essential amino acids for targeted animal species.

Current research indicates a future role for macroalgae in the sustainable production of formulated compound feed that will improve animal health. There is increasing interest in the potentially beneficial effects of the variety of bioactive compounds in macroalgae, such as laminarin, fucoidan, and phlorotannins. Future commercialization will benefit from biorefinery approaches to developing cost‐effective and environmentally‐friendly extraction methods to produce interesting bioactive compounds with quantified beneficial effects. In this context, more research is needed to evaluate nutritional properties and mechanisms underlying the health benefits of a wide variety of macroalgal products intended for terrestrial and aquacultural monogastric animals.

## References

[1] Godfray HCJ , Beddington JR , Crute IR , Haddad L , Lawrence D , Muir JF *et al*, Food security: the challenge of feeding 9 billion people. Science 327:812–818 (2010). 20110467 10.1126/science.1185383

[2] Boland MJ , Rae AN , Vereijken JM , Meuwissen MPM , Fischer ARH , van Boekel M *et al*, The future supply of animal‐derived protein for human consumption. Trends Food Sci Technol 29:62–73 (2013).

[3] Evans FD and Critchley AT , Seaweeds for animal production use. J Appl Phycol 26:891–899 (2014).

[4] Angell AR , Angell SF , de Nys R and Paul NA , Seaweed as a protein source for mono‐gastric livestock. Trends Food Sci Technol 54:74–84 (2016).

[5] Garcia‐Vaquero M and Hayes M , Red and green macroalgae for fish and animal feed and human functional food development. Food Rev Int 32:15–45 (2016).

[6] Makkar HPS , Tran G , Heuze V , Giger‐Reverdin S , Lessire M , Lebas F *et al*, Seaweeds for livestock diets: a review. Anim Feed Sci Technol 212:1–17 (2016).

[7] Jensen A , Present and future needs for algae and algal products. Hydrobiologia 261:15–23 (1993).

[8] Patarra R , Paiva L , Neto AI , Lima E and Baptista J , Nutritional value of selected macroalgae. J Appl Phycol 23:205–208 (2011).

[9] Fleurence J , Morancais M , Dumay J , Decottignies P , Turpin V , Munier M *et al*, What are the prospects for using seaweed in human nutrition and for marine animals raised through aquaculture? Trends Food Sci Technol 27:57–61 (2012).

[10] Mæhre HK , Malde MK , Eilertsen KE and Elvevoll EO , Characterization of protein, lipid and mineral contents in common Norwegian seaweeds and evaluation of their potential as food and feed. J Sci Food Agric 94:3281–3290 (2014). 24700148 10.1002/jsfa.6681

[11] Nielsen MM , Manns D , D'Este M , Krause‐Jensen D , Rasmussen MB , Larsen MM *et al*, Variation in biochemical composition of *Saccharina latissima* and *Laminaria digitata* along an estuarine salinity gradient in inner Danish waters. Algal Res 13:235–245 (2016).

[12] Kumar S , Sahoo D and Levine I , Assessment of nutritional value in a brown seaweed *Sargassum wightii* and their seasonal variations. Algal Res 9:117–125 (2015).

[13] MacArtain P , Gill CIR , Brooks M , Campbell R and Rowland IR , Nutritional value of edible seaweeds. Nutr Rev 65:535–543 (2007). 18236692 10.1301/nr.2007.dec.535-543

[14] Gupta S and Abu‐Ghannam N , Bioactive potential and possible health effects of edible brown seaweeds. Trends Food Sci Technol 22:315–326 (2011).

[15] Mohamed S , Hashim SN and Rahman HA , Seaweeds: a sustainable functional food for complementary and alternative therapy. Trends Food Sci Technol 23:83–96 (2012).

[16] Yu P and Gu HF , Bioactive substances from marine fishes, shrimps, and algae and their functions: present and future. Crit Rev Food Sci Nutr 55:1112–1134 (2015). 10.1080/10408398.2012.686933 24915345

[17] Corona G , Ji Y , Anegboonlap P , Hotchkiss S , Gill C , Yaqoob P *et al*, Gastrointestinal modifications and bioavailability of brown seaweed phlorotannins and effects on inflammatory markers. Br J Nutr 115:1240–1253 (2016). 26879487 10.1017/S0007114516000210

[18] Kadam SU , Tiwari BK and O'Donnell CP , Application of novel extraction technologies for bioactives from marine algae. J Agric Food Chem 61:4667–4675 (2013). 23634989 10.1021/jf400819p

[19] Schiener P , Stanley MS , Black KD and Green DH , Assessment of saccharification and fermentation of brown seaweeds to identify the seasonal effect on bioethanol production. J Appl Phycol 28:3009–3020 (2016).

[20] Boderskov T , Schmedes PS , Bruhn A , Rasmussen MB , Nielsen MM and Pedersen MF , The effect of light and nutrient availability on growth, nitrogen, and pigment contents of *Saccharina latissima* (Phaeophyceae) grown in outdoor tanks, under natural variation of sunlight and temperature, during autumn and early winter in Denmark. J Appl Phycol 28:1153–1165 (2016).

[21] Mortensen LM , Remediation of nutrient‐rich, brackish fjord water through production of protein‐rich kelp *S. latissima* and *L. digitata* . J Appl Phycol 29:3089–3096 (2017).

[22] Sharma S , Neves L , Funderud J , Mydland LT and Øverland M , Seasonal and depth variations in the chemical composition of cultivated *Saccharina latissima* . Algal Res 32:107–112 (2018).

[23] Marinho GS , Holdt SL and Angelidaki I , Seasonal variations in the amino acid profile and protein nutritional value of *Saccharina latissima* cultivated in a commercial IMTA system. J Appl Phycol 27:1991–2000 (2015).

[24] Verma P , Kumar M , Mishra G and Sahoo D , Multivariate analysis of fatty acid and biochemical constitutes of seaweeds to characterize their potential as bioresource for biofuel and fine chemicals. Bioresour Technol 226:132–144 (2017). 27997867 10.1016/j.biortech.2016.11.044

[25] Belghit I , Rasinger JD , Heesch S , Biancarosa I , Liland N , Torstensen B *et al*, In‐depth metabolic profiling of marine macroalgae confirms strong biochemical differences between brown, red and green algae. Algal Res 26:240–249 (2017).

[26] Holdt SL and Kraan S , Bioactive compounds in seaweed: functional food applications and legislation. J Appl Phycol 23:543–597 (2011).

[27] Pereira CMP , Nunes CFP , Zambotti‐Villela L , Streit NM , Dias D , Pinto E *et al*, Extraction of sterols in brown macroalgae from Antarctica and their identification by liquid chromatography coupled with tandem mass spectrometry. J Appl Phycol 29:751–757 (2017).

[28] van den Burg SWK , Stuiver M , Veenstra FA , Bikker P , Lopez Contreras AM , Palstra AP , Broeze J , Jansen HM , Jak RG , Gerritsen AL , Harmsen PFH , Kals J , Blanco Garcia A , Brandenburg WA , van Krimpen MM , van Duijn AP , Mulder WJ and van Raamsdonk LWD , A Triple P review of the feasibility of sustainable offshore seaweed production in the North Sea. LEI report 13‐077, Wageningen UR (2013).

[29] Tibbetts SM , Milley JE and Lall SP , Nutritional quality of some wild and cultivated seaweeds: nutrient composition, total phenolic content and *in vitro* digestibility. J Appl Phycol 28:3575–3585 (2016).

[30] Rohani‐Ghadikolaei K , Abdulalian E and Ng W‐K , Evaluation of the proximate, fatty acid and mineral composition of representative green, brown and red seaweeds from the Persian Gulf of Iran as potential food and feed resources. J Food Sci Technol 49:774–780 (2012). 24293698 10.1007/s13197-010-0220-0 PMC3550831

[31] Smith JL , Summers G and Wong R , Nutrient and heavy metal content of edible seaweeds in New Zealand. N Z J Crop Hortic Sci 38:19–28 (2010).

[32] Angell AR , Mata L , de Nys R and Paul NA , The protein content of seaweeds: a universal nitrogen‐to‐protein conversion factor of five. J Appl Phycol 28:511–524 (2016).

[33] Lowry OH , Rosebrough NJ , Farr AL and Randall RJ , Protein measurement with the Folin phenol reagent. J Biol Chem 193:265–275 (1951). 14907713

[34] Bradford MM , A rapid and sensitive method for the quantitation of microgram quantities of protein utilizing the principle of protein‐dye binding. Anal Biochem 72:248–254 (1976). 942051 10.1016/0003-2697(76)90527-3

[35] Lourenco SO , Barbarino E , De‐Paula JC , Pereira LO and Marquez UML , Amino acid composition, protein content and calculation of nitrogen‐to‐protein conversion factors for 19 tropical seaweeds. Phycol Res 50:233–241 (2002).

[36] Dawczynski C , Schubert R and Jahreis G , Amino acids, fatty acids, and dietary fibre in edible seaweed products. Food Chem 103:891–899 (2007).

[37] Biancarosa I , Espe M , Bruckner CG , Heesch S , Liland N , Waagbo R *et al*, Amino acid composition, protein content, and nitrogen‐to‐protein conversion factors of 21 seaweed species from Norwegian waters. J Appl Phycol 29:1001–1009 (2017).

[38] Bogolitsyn KG , Kaplitsin PA and Pochtovalova AS , Amino‐acid Composition of Arctic Brown algae. Chem Nat Compd 49:1110–1113 (2014).

[39] Ortiz J , Romero N , Robert P , Araya J , Lopez‐Hernandez J , Bozzo C *et al*, Dietary fiber, amino acid, fatty acid and tocopherol contents of the edible seaweeds *Ulva lactuca* and *Durvillaea antarctica* . Food Chem 99:98–104 (2006).

[40] Mišurcová L , Bunka F , Ambrozova JV , Machu L , Samek D and Kracmar S , Amino acid composition of algal products and its contribution to RDI. Food Chem 151:120–125 (2014). 24423510 10.1016/j.foodchem.2013.11.040

[41] Bikker P , van Krimpen MM , van Wikselaar P , Houweling‐Tan B , Scaccia N , van Hal JW *et al*, Biorefinery of the green seaweed *Ulva lactuca* to produce animal feed, chemicals and biofuels. J Appl Phycol 28:3511–3525 (2016). 28035175 10.1007/s10811-016-0842-3 PMC5155021

[42] Martone PT , Estevez JM , Lu FC , Ruel K , Denny MW , Somerville C *et al*, Discovery of lignin in seaweed reveals convergent evolution of cell‐wall architecture. Curr Biol 19:169–175 (2009). 19167225 10.1016/j.cub.2008.12.031

[43] Weng JK and Chapple C , The origin and evolution of lignin biosynthesis. New Phytol 187:273–285 (2010). 20642725 10.1111/j.1469-8137.2010.03327.x

[44] Florez N , Gonzalez‐Munoz MJ , Ribeiro D , Fernandes E , Dominguez H and Freitas M , Algae Polysaccharides' chemical characterization and their role in the inflammatory process. Curr Med Chem 24:149–175 (2017). 27804878 10.2174/0929867323666161028160416

[45] Raposo MFD , de Morais AMB and de Morais R , Marine polysaccharides from algae with potential biomedical applications. Mar Drugs 13:2967–3028 (2015). 25988519 10.3390/md13052967 PMC4446615

[46] Senni K , Pereira J , Gueniche F , Delbarre‐Ladrat C , Sinquin C , Ratiskol J *et al*, Marine polysaccharides: a source of bioactive molecules for cell therapy and tissue engineering. Mar Drugs 9:1664–1681 (2011). 22131964 10.3390/md9091664 PMC3225941

[47] Jiao GL , Yu GL , Zhang JZ and Ewart HS , Chemical structures and bioactivities of sulfated polysaccharides from marine algae. Mar Drugs 9:196–223 (2011). 21566795 10.3390/md9020196 PMC3093253

[48] Cian RE , Drago SR , de Medina FS and Martinez‐Augustin O , Proteins and carbohydrates from red seaweeds: evidence for beneficial effects on gut function and microbiota. Mar Drugs 13:5358–5383 (2015). 26308006 10.3390/md13085358 PMC4557026

[49] Lahaye M , Marine‐algae as sources of fibers ‐ determination of soluble and insoluble dietary fiber contents in some sea vegetables. J Sci Food Agric 54:587–594 (1991).

[50] Venkatesan J , Lowe B , Anil S , Manivasagan P , Al Kheraif AA , Kang KH *et al*, Seaweed polysaccharides and their potential biomedical applications. Starch‐Starke 67:381–390 (2015).

[51] Wells ML , Potin P , Craigie JS , Raven JA , Merchant SS , Helliwell KE *et al*, Algae as nutritional and functional food sources: revisiting our understanding. J Appl Phycol 29:949–982 (2017). 28458464 10.1007/s10811-016-0974-5 PMC5387034

[52] Percival E , The polysaccharides of green, red and brown seaweeds: their basic structure, biosynthesis and function. Br Phycol J 14:103–117 (1979).

[53] Jung KA , Lim SR , Kim Y and Park JM , Potentials of macroalgae as feedstocks for biorefinery. Bioresour Technol 135:182–190 (2013). 23186669 10.1016/j.biortech.2012.10.025

[54] Garcia‐Vaquero M , Rajauria G , O'Doherty JV and Sweeney T , Polysaccharides from macroalgae: recent advances, innovative technologies and challenges in extraction and purification. Food Res Int 99:1011–1020 (2017). 28865611 10.1016/j.foodres.2016.11.016

[55] Raposo MFD , de Morais A and de Morais R , Emergent sources of prebiotics: seaweeds and microalgae. Mar Drugs 14:14–27 (2016). 26828501 10.3390/md14020027 PMC4771980

[56] Chopin T , Sharp G , Belyea E , Semple R and Jones D , Open‐water aquaculture of the red alga Chondrus crispus in Prince Edward Island, Canada. Hydrobiologia 399:417–425 (1999).

[57] Jimenez‐Escrig A and Sanchez‐Muniz FJ , Dietary fibre from edible seaweeds: chemical structure, physicochemical properties and effects on cholesterol metabolism. Nutr Res 20:585–598 (2000).

[58] van Ginneken VJT , Helsper J , de Visser W , van Keulen H and Brandenburg WA , Polyunsaturated fatty acids in various macroalgal species from North Atlantic and tropical seas. Lipids Health Dis 10:8 (2011). 21696609 10.1186/1476-511X-10-104 PMC3131239

[59] Santos MAZ , Colepicolo P , Pupo D , Fujii MT , de Pereira CMP and Mesko MF , Antarctic red macroalgae: a source of polyunsaturated fatty acids. J Appl Phycol 29:759–767 (2017).

[60] Pereira H , Barreira L , Figueiredo F , Custodio L , Vizetto‐Duarte C , Polo C *et al*, Polyunsaturated fatty acids of marine macroalgae: potential for nutritional and pharmaceutical applications. Mar Drugs 10:1920–1935 (2012). 23118712 10.3390/md10091920 PMC3475264

[61] Perez MJ , Falque E and Dominguez H , Antimicrobial action of compounds from marine seaweed. Mar Drugs 14:14–42 (2016). 27005637 10.3390/md14030052 PMC4820306

[62] Almela C , Clemente MJ , Velez D and Montoro R , Total arsenic, inorganic arsenic, lead and cadmium contents in edible seaweed sold in Spain. Food Chem Toxicol 44:1901–1908 (2006). 16901603 10.1016/j.fct.2006.06.011

[63] Herrmann C , FitzGerald J , O'Shea R , Xia A , O'Kiely P and Murphy JD , Ensiling of seaweed for a seaweed biofuel industry. Bioresour Technol 196:301–313 (2015). 26253914 10.1016/j.biortech.2015.07.098

[64] Black WAP , The preservation of seaweed by ensiling and bactericides. J Sci Food Agric 6:14–23 (1955).

[65] Uchida M and Miyoshi T , Algal fermentation‐the seed for a new fermentation industry of foods and related products. Jpn Agric Res Q 47:53–63 (2013).

[66] Gallagher JA , Turner LB , Adams JMM , Dyer PW and Theodorou MK , Dewatering treatments to increase dry matter content of the brown seaweed, kelp (*Laminaria digitata* ((Hudson) JV Lamouroux)). Bioresour Technol 224:662–669 (2017). 27956334 10.1016/j.biortech.2016.11.091

[67] McHugh D , *A guide to the seaweed industry*. FAO fish tech pap, FAO, in Rome, Italy. (2003).

[68] Chan JCC , Cheung PCK and Ang PO , Comparative studies on the effect of three drying methods on the nutritional composition of seaweed *Sargassum hemiphyllum* (Turn) C ag. J Agric Food Chem 45:3056–3059 (1997).

[69] Fleurence J , LeCoeur C , Mabeau S , Maurice M and Landrein A , Comparison of different extractive procedures for proteins from the edible seaweeds *Ulva rigida* and *Ulva rotundata* . J Appl Phycol 7:577–582 (1995).

[70] Wong KH and Cheung PCK , Nutritional evaluation of some subtropical red and green seaweeds Part II. *In vitro* protein digestibility and amino acid profiles of protein concentrates. Food Chem 72:11–17 (2001).

[71] Barbarino E and Lourenco SO , An evaluation of methods for extraction and quantification of protein from marine macro‐ and microalgae. J Appl Phycol 17:447–460 (2005).

[72] Joubert Y and Fleurence J , Simultaneous extraction of proteins and DNA by an enzymatic treatment of the cell wall of *Palmaria palmata* (Rhodophyta). J Appl Phycol 20:55–61 (2008).

[73] Bleakley S and Hayes M , Algal proteins: extraction, application, and challenges concerning production. Foods 6:33 (2017). 10.3390/foods6050033 PMC5447909 28445408

[74] Harnedy PA and FitzGerald RJ , Bioactive proteins, peptides, and amino acids from macroalgae. J Phycol 47:218–232 (2011). 27021854 10.1111/j.1529-8817.2011.00969.x

[75] Marrion O , Schwertz A , Fleurence J , Gueant JL and Villaume C , Improvement of the digestibility of the proteins of the red alga *Palmaria palmata* by physical processes and fermentation. Nahrung‐Food 47:339–344 (2003). 10.1002/food.200390078 14609091

[76] Adams JM , Gallagher JA and Donnison IS , Fermentation study on *Saccharina latissima* for bioethanol production considering variable pre‐treatments. J Appl Phycol 21:569–574 (2009).

[77] Manns D , Andersen SK , Saake B and Meyer AS , Brown seaweed processing: enzymatic saccharification of *Laminaria digitata* requires no pre‐treatment. J Appl Phycol 28:1287–1294 (2016).

[78] Sharma S and Horn SJ , Enzymatic saccharification of brown seaweed for production of fermentable sugars. Bioresour Technol 213:155–161 (2016). 26961713 10.1016/j.biortech.2016.02.090

[79] Øverland M and Skrede A , Yeast derived from lignocellulosic biomass as a sustainable feed resource for use in aquaculture. J Sci Food Agric 97:733–742 (2017). 27558451 10.1002/jsfa.8007

[80] Nielsen MM , Bruhn A , Rasmussen MB , Olesen B , Larsen MM and Moller HB , Cultivation of *Ulva lactuca* with manure for simultaneous bioremediation and biomass production. J Appl Phycol 24:449–458 (2012).

[81] Abudabos AM , Okab AB , Aljumaah RS , Samara EM , Abdoun KA and Al‐Haidary AA , Nutritional value of green seaweed (*Ulva lactuca*) for broiler chickens. Ital J Anim Sci 12:e28.177–181 (2013). 10.2527/jas.2013-6719 24146153

[82] Wang S , Shi X , Zhou C and Lin Y , *Enteromorpha prolifera*: effects on performance, carcass quality and small intestinal digestive enzyme activities of broilers. Chin J Anim Nutr 25:1332–1337 (2013).

[83] Ventura MR , Castanon JIR and McNab JM , Nutritional value of seaweed (*Ulva rigida*) for poultry. Anim Feed Sci Technol 49:87–92 (1994).

[84] Valente LMP , Gouveia A , Rema P , Matos J , Gomes EF and Pinto IS , Evaluation of three seaweeds *Gracilaria bursa‐pastoris*, *Ulva rigida* and *Gracilaria cornea* as dietary ingredients in European sea bass (*Dicentrarchus labrax*) juveniles. Aquaculture 252:85–91 (2006).

[85] Marinho G , Nunes C , Sousa‐Pinto I , Pereira R , Rema P and Valente LMP , The IMTA‐cultivated Chlorophyta *Ulva* spp. as a sustainable ingredient in Nile tilapia (*Oreochromis*) diets. J Appl Phycol 25:1359–1367 (2013).

[86] Wassef EA , El‐Sayed AFM , Kandeel KA and Sakr EM , Evaluation of *Pterocl dia* (Rodophyta) and *Ulva* (Chlorophyta) meals as additives to gilthead seabream *Sparus aurata* diets. Egypt J Aqua Res 31:321–332 (2005).

[87] Mustafa MG , Wakamatsu S , Takeda T and Umino T , Effects of algae meal as feed additive on growth, feed efficiency, and body composition in red sea bream. Fish Sci 61:25–28 (1995).

[88] Marrion O , Fleurence J , Schwertz A , Gueant JL , Mamelouk L , Ksouri J *et al*, Evaluation of protein *in vitro* digestibility of *Palmaria palmata* and *Gracilaria verrucosa* . J Appl Phycol 17:99–102 (2005).

[89] Maehre HK , Edvinsen GK , Eilertsen KE and Elvevoll EO , Heat treatment increases the protein bioaccessibility in the red seaweed dulse (*Palmaria palmata*), but not in the brown seaweed winged kelp (*Alaria esculenta*). J Appl Phycol 28:581–590 (2016).

[90] Wan AHL , Soler‐Vila A , O'Keeffe D , Casburn P , Fitzgerald R and Johnson MP , The inclusion of *Palmaria palmata* macroalgae in Atlantic salmon (*Salmo salar*) diets: effects on growth, haematology, immunity and liver function. J Appl Phycol 28:3091–3100 (2016).

[91] Moroney NC , Wan AHL , Soler‐Vila A , FitzGerald RD , Johnson MP and Kerry JP , Inclusion of *Palmaria palmata* (red seaweed) in Atlantic salmon diets: effects on the quality, shelf‐life parameters and sensory properties of fresh and cooked salmon fillets. J Sci Food Agric 95:897–905 (2015). 24852938 10.1002/jsfa.6753

[92] Davies S , Brown M and Camilleri M , Preliminary assessment of the seaweed *Porphyra purpurea* in artificial diets for the tick‐lipped grey mullet (*Chelon labrosus*). Aquaculture 152:249–258 (1997).

[93] Walker AB , Fournier HR , Neffus CD , Nardi GC and Berlinsky DL , Partial replacement of fish meal with laver *Porphyra* spp. in diets for Atlantic cod. N Am J Aquacult 71:39–45 (2009).

[94] Soler‐Vila A , Coughlan S , Guiry MD and Kraan S , The red alga *Porphyra dioica* as a fish‐feed ingredient for rainbow trout (*Oncorhynchus mykiss*): effects on growth, feed efficiency, and carcass composition. J Appl Phycol 21:617–624 (2009).

[95] Araujo M , Rema P , Sousa‐Pinto I , Cunha LM , Peixoto MJ , Pires MA *et al*, Dietary inclusion of IMTA‐cultivated *Gracilaria vermiculophylla* in rainbow trout (*Oncorhynchus mykiss*) diets: effects on growth, intestinal morphology, tissue pigmentation, and immunological response. J Appl Phycol 28:679–689 (2016).

[96] Sotoudeh E and Mardani F , Antioxidant‐related parameters, digestive enzyme activity and intestinal morphology in rainbow trout (*Oncorhynchus mykiss*) fry fed graded levels of red seaweed, *Gracilaria pygmaea* . Aquacult Nutr 24:777–785 (2018).

[97] Peixoto MJ , Svendsen JC , Malte H , Pereira LF , Carvalho P , Pereira R *et al*, Diets supplemented with seaweed affect metabolic rate, innate immune, and antioxidant responses, but not individual growth rate in European seabass (*Dicentrarchus labrax*). J Appl Phycol 28:2061–2071 (2016).

[98] Stadtlander T , Khalil WKB , Focken U and Becker K , Effects of low and medium levels of red alga Nori (*Porphyra yezoensis Ueda*) in the diets on growth, feed utilization and metabolism in intensively fed Nile tilapia, *Oreochromis niloticus* (L.). Aquacult Nutr 19:64–73 (2013).

[99] Jones RT , Blunden C and Probert AJ , Effects of dietary *Ascophyllum nodosum* on blood parameters of rats and pigs. Bot Marina 22:393–402 (1979).

[100] Whittemore CT and Percival JK , A seaweed residue unsuitable as a major source of energy or nitrogen for growing pigs. J Sci Food Agric 26:215–217 (1975). 1134059 10.1002/jsfa.2740260212

[101] Costa MM , Oliveira STL , Balen RE , Bueno Junior G , Baldan LT , Silva LCR *et al*, Brown seaweed meal to Nile tilapia fingerlings. Arch Zootec 62:101–109 (2013).

[102] Moroney NC , O'Grady MN , O'Doherty JV and Kerry JP , Addition of seaweed (*Laminaria digitata*) extracts containing laminarin and fucoidan to porcine diets: influence on the quality and shelf‐life of fresh pork. Meat Sci 92:423–429 (2012). 22673179 10.1016/j.meatsci.2012.05.005

[103] Eom SH , Kim YM and Kim SK , Antimicrobial effect of phlorotannins from marine brown algae. Food Chem Toxicol 50:3251–3255 (2012). 22735502 10.1016/j.fct.2012.06.028

[104] Gardiner GE , Campbell AJ , O'Doherty JV , Pierce E , Lynch PB , Leonard FC *et al*, Effect of *Ascophyllum nodosum e*xtract on growth performance, digestibility, carcass characteristics and selected intestinal microflora populations of grower‐finisher pigs. Anim Feed Sci Technol 141:259–273 (2008).

[105] McDonnell P , Figat S and O'Doherty JV , The effect of dietary laminarin and fucoidan in the diet of the weanling piglet on performance, selected faecal microbial populations and volatile fatty acid concentrations. Animal 4:579–585 (2010). 22444045 10.1017/S1751731109991376

[106] O'Doherty JV , Dillon S , Figat S , Callan JJ and Sweeney T , The effects of lactose inclusion and seaweed extract derived from *Laminaria* spp. on performance, digestibility of diet components and microbial populations in newly weaned pigs. Anim Feed Sci Technol 157:173–180 (2010).

[107] Leonard SG , Sweeney T , Bahar B , Lynch BP and O'Doherty JV , Effects of dietary seaweed extract supplementation in sows and post‐weaned pigs on performance, intestinal morphology, intestinal microflora and immune status. Br J Nutr 106:688–699 (2011). 21736851 10.1017/S0007114511000997

[108] Leonard SG , Sweeney T , Bahar B , Lynch BP and O'Doherty JV , Effect of dietary seaweed extracts and fish oil supplementation in sows on performance, intestinal microflora, intestinal morphology, volatile fatty acid concentrations and immune status of weaned pigs. Br J Nutr 105:549–560 (2011). 20875191 10.1017/S0007114510003739

[109] Walsh AM , Sweeney T , O'Shea CJ , Doyle DN and O 'Doherty JV , Effect of supplementing varying inclusion levels of laminarin and fucoidan on growth performance, digestibility of diet components, selected faecal microbial populations and volatile fatty acid concentrations in weaned pigs. Anim Feed Sci Technol 183:151–159 (2013).

[110] Walsh AM , Sweeney T , O'Shea CJ , Doyle DN and O'Doherty JV , Effect of dietary laminarin and fucoidan on selected microbiota, intestinal morphology and immune status of the newly weaned pig. Br J Nutr 110:1630–1638 (2013). 23531383 10.1017/S0007114513000834

[111] O'Shea CJ , McAlpine P , Sweeney T , Varley PF and O'Doherty JV , Effect of the interaction of seaweed extracts containing laminarin and fucoidan with zinc oxide on the growth performance, digestibility and faecal characteristics of growing piglets. Br J Nutr 111:798–807 (2014). 24131869 10.1017/S0007114513003280

[112] Heim G , Walsh AM , Sweeney T , Doyle DN , O'Shea CJ , Ryan MT *et al*, Effect of seaweed‐derived laminarin and fucoidan and zinc oxide on gut morphology, nutrient transporters, nutrient digestibility, growth performance and selected microbial populations in weaned pigs. Br J Nutr 111:1577–1585 (2014). 24502994 10.1017/S0007114513004224

[113] Bouwhuis MA , Sweeney T , Mukhopadhya A , McDonnell MJ and O'Doherty JV , Maternal laminarin supplementation decreases *Salmonella* Typhimurium shedding and improves intestinal health in piglets following an experimental challenge with *S*.Typhimurium post‐weaning. Anim Feed Sci Technol 223:156–168 (2017).

[114] Sweeney T , Meredith H , Ryan MT , Gath V , Thornton K and O'Doherty JV , Effects of *Ascophyllum nodosum* supplementation on *Campylobacter jejuni* colonisation, performance and gut health following an experimental challenge in 10 day old chicks. Innov Food Sci Emerg Technol 37:247–252 (2016).

[115] Sweeney T , Meredith H , Vigors S , McDonnell MJ , Ryan M , Thornton K *et al*, Extracts of laminarin and laminarin/fucoidan from the marine macroalgal species *Laminaria digitata* improved growth rate and intestinal structure in young chicks, but does not influence *Campylobacter jejuni* colonisation. Anim Feed Sci Technol 232:71–79 (2017).

[116] Vatsos IN and Rebours C , Seaweed extracts as antimicrobial agents in aquaculture. J Appl Phycol 27:2017–2035 (2015).

[117] Lynch MB , Sweeney T , Callan JJ , O'Sullivan JT and O'Doherty JV , The effect of dietary *Laminaria*‐derived laminarin and fucoidan on nutrient digestibility, nitrogen utilisation, intestinal microflora and volatile fatty acid concentration in pigs. J Sci Food Agric 90:430–437 (2010). 20355064 10.1002/jsfa.3834

[118] Reilly P , O'Doherty JV , Pierce KM , Callan JJ , O'Sullivan JT and Sweeney T , The effects of seaweed extract inclusion on gut morphology, selected intestinal microbiota, nutrient digestibility, volatile fatty acid concentrations and the immune status of the weaned pig. Animal 2:1465–1473 (2008). 22443904 10.1017/S1751731108002711

[119] Gahan DA , Lynch MB , Callan JJ , O'Sullivan JT and O'Doherty JV , Performance of weanling piglets offered low‐, medium‐ or high‐lactose diets supplemented with a seaweed extract from *Laminaria* spp. Animal 3:24–31 (2009). 22444169 10.1017/S1751731108003017

[120] Sweeney T , Collins CB , Reilly P , Pierce KM , Ryan M and O'Doherty JV , Effect of purified beta‐glucans derived from *Laminaria digitata*, *Laminaria hyperborea* and *Saccharomyces cerevisiae* on piglet performance, selected bacterial populations, volatile fatty acids and pro‐inflammatory cytokines in the gastrointestinal tract of pigs. Br J Nutr 108:1226–1234 (2012). 22313684 10.1017/S0007114511006751

[121] Ryan MT , Smith AG , O'Doherty JV , Bahar B , Reilly P and Sweeney T , Effects of nutrient supplementation with laminarin derived from Laminaria hyperborea and Laminaria digitata on mucin gene expression in the porcine ileum. Livest Sci 133:236–238 (2010).

[122] Leonard SG , Sweeney T , Bahar B and O'Doherty JV , Effect of maternal seaweed extract supplementation on suckling piglet growth, humoral immunity, selected microflora, and immune response after an *ex vivo* lipopolysaccharide challenge. J Anim Sci 90:505–514 (2012). 21948611 10.2527/jas.2010-3243

[123] Turner JL , Dritz SS , Higgins JJ and Minton JE , Effects of *Ascophyllum nodosum* extract on growth performance and immune function of young pigs challenged with *Salmonella typhimurium* . J Anim Sci 80:1947–1953 (2002). 12162664 10.2527/2002.8071947x

[124] Michiels J , Skrivanova E , Missotten J , Ovyn A , Mrazek J , De Smet S *et al*, Intact brown seaweed (*Ascophyllum nodosum*) in diets of weaned piglets: effects on performance, gut bacteria and morphology and plasma oxidative status. J Anim Physiol Anim Nutr 96:1101–1111 (2012). 10.1111/j.1439-0396.2011.01227.x 21917025

[125] Dierick N , Ovyn A and De Smet S , Effect of feeding intact brown seaweed *Ascophyllum nodosum* on some digestive parameters and on iodine content in edible tissues in pigs. J Sci Food Agric 89:584–594 (2009).

[126] Bansemir A , Blume M , Schroder S and Lindequist U , Screening of cultivated seaweeds for antibacterial activity against fish pathogenic bacteria. Aquaculture 252:79–84 (2006).

[127] Valente LMP , Araujo M , Batista S , Peixoto MJ , Sousa‐Pinto I , Brotas V *et al*, Carotenoid deposition, flesh quality and immunological response of Nile tilapia fed increasing levels of IMTA‐cultivated *Ulva* spp. J Appl Phycol 28:691–701 (2016).

[128] Wassef EA , El‐Sayed AFM and Sakr EM , *Pterocladia* (Rhodophyta) and *Ulva* (Chlorophyta) as feed supplements for European seabass, *Dicentrarchus labrax* L., fry. J Appl Phycol 25:1369–1376 (2013).

[129] Yin GW , Li WW , Lin Q , Lin X , Lin JB , Zhu QG *et al*, Dietary administration of laminarin improves the growth performance and immune responses in *Epinephelus coioides* . Fish Shellfish Immunol 41:402–406 (2014). 25266890 10.1016/j.fsi.2014.09.027

